# Molecular Identification of *Fusarium* Isolates from Bozcaada Çavuş and Karalahna Grapes in Türkiye

**DOI:** 10.3390/jof11050373

**Published:** 2025-05-14

**Authors:** Gülçin Özcan Ateş

**Affiliations:** Department of Medical Services and Techniques, Health Services Vocational School, Çanakkale Onsekiz Mart University, Çanakkale 17020, Türkiye; gulcinozcan87@gmail.com or gulcinozcan@comu.edu.tr

**Keywords:** *Fusarium*, *tef1*, βeta-tubulin, Çavuş grape, Karalahna grape

## Abstract

Contamination of agricultural products by *Fusarium* species is a significant concern and is commonly found in various agricultural products. They cause severe economic losses in the products, and contaminate and threaten human and animal health due to the toxins they produce. Therefore, determining species diversity in various agricultural products is crucial. Bozcaada is well suited for cultivating the highest quality Çavuş grape due to its unique location and climate. Therefore, in this study, the sequencing of the *tef1* and *tub2* genes in *Fusarium* isolates from table Çavuş and wine grapes Karalahna, which are specific to Bozcaada, was performed, and their phylogenetic relationships were examined. As a result, it was determined that 11 of the 17 isolates were *Fusarium annulatum* from the *Fusarium fujikuroi* species complex (FFSC), 2 were *Fusarium nirenbergiae* from the *Fusarium oxysporum* species complex (FOSC), 2 were *Fusarium fabacearum* from the FOSC, and the last 2 isolates were *Fusarium makinsoniae* and *Fusarium clavus* (as ‘*clavum’*) from the *F. incarnatum-equiseti* species complex (FIESC). The *F. makinsoniae* and *F. fabacearum* species obtained in the study are the first recorded for Türkiye. This research highlights the variety of *Fusarium* species identified in Bozcaada vineyards in Türkiye.

## 1. Introduction

Bozcaada is located at the exit of the Çanakkale Strait and in the northeast of the Aegean Sea, formed by the breaking and collapse of the earth’s crust approximately 2–2.5 million years ago at the end of the III Geological Period. Bozcaada has an area of approximately 36 km^2^ and is the third largest island in Türkiye. Under the influence of the Mediterranean and continental climates, the island is dominated by maquis vegetation and has the typical flora of Western Anatolia. Due to its unique location and climate, it has been identified with viticulture and winemaking throughout history. The island’s low and flat topographic structure and the climate conditions, with northerly and southerly winds due to its location right at the exit of a strait, result in unique aromas for the grape varieties grown here [1,2]. Therefore, the island’s most significant source of income is viticulture, and table Çavuş grapes and the wine grape varieties Karalahna, Kuntra, and Vasilaki are grown [3,4]. Due to the island’s climate and soil structure, the world’s most delicious and high-quality table Çavuş grape is grown on the island, and it has a thin skin and a unique aroma. Due to these proven superior features of Bozcaada Çavuş Grape, it received a Geographical Indication Registration Certificate in 2020. The name “Bozcaada Çavuşu Grape” has become an essential part of the region’s viticultural heritage [4,5]. The wine Karalahna grape, which is specific to Bozcaada, is a dark purple, thick-skinned, round, and large-grained variety, with a colorless and fleshy interior. The Karalahna grape, which attracts attention with its dark ruby color and balanced acidity, has been used for many years as a source in the color improvement of light-colored wines to enhance their color. Today, it is primarily used to improve wine quality. Again, although the unique ecological characteristics of the island contribute to the Karalahna grape variety acquiring distinct characteristics, unlike the Çavuş grape, there is limited information in the literature about the characteristics of the Karalahna grape variety [6,7].

High water and sugar contents, combined with low pH values, create a suitable environment for mold to develop in fruits. For this reason, mold-related spoilage is observed in fruits. Some molds are phytopathogenic, affecting the leaves, shoots, stems, and fruits of plants, and can cause the spoilage of the product before it is harvested. Others can cause spoilage both before and after harvest. Fungal diseases, observed both before and after harvest, primarily affect the quality of the fruit and, consequently, the quality of the product, resulting in severe economic losses. Grapes are a seasonal fruit with a short storage life. Fungal spoilage in grapes reduces both the yield and quality of the grapes. Fungal spoilage in grapes is seen especially during the storage period. *Botrytis*, *Rhizopus*, and *Mucor* mold species damage fruits and cause soft rot. Toxigenic or pathogenic species, such as *Aspergillus* spp. and *Penicillium* spp., that develop during storage cause a decrease in grape quality and pose a health risk to consumers. While the presence of pathogenic molds can cause allergies in susceptible individuals, mycotoxins formed by toxigenic species pose serious health risks [8,9,10].

*Fusarium* species are ubiquitous, hyaline filamentous molds with a cosmopolitan distribution. They belong to the Nectriaceae family within the Hypocreales order of the Ascomycota fungal phylum. Most *Fusarium* species are soil-borne and can be found in the air, water, plants, insects, soil, and organic substrates. They exhibit endophytic, saprophytic, hemi-biotrophic, or parasitic forms and possess strong competitive abilities. Based on host relationships, morphology, and molecular characterizations, the genus *Fusarium* is estimated to include more than 400 recognized species, grouped into 23 species complexes. They are among the most destructive plant pathogens and mycotoxigenic microfungi, causing significant reductions in crop yields across various agricultural crops, resulting in multi-billion-dollar losses. Key plant diseases attributed to them include head blight in cereals, root rot in peas, ear rot in corn, sudden death syndrome in soybeans, and vascular wilt in various crops. The two most important species affecting plants are *Fusarium graminearum* and *Fusarium oxysporum*, which rank among the top five phytopathogens [9,11,12,13].

The risk of the fungal contamination of grapes with mycotoxin-producing *Aspergillus*, *Alternaria*, and *Penicillium* species is a well-known fact. Regarding microfungi and metabolites that may affect human health in grapes and their products, studies in the literature have focused particularly on *Aspergillus* spp. contamination and ochratoxin A (OTA) production [2,14,15,16,17,18,19]. *Aspergillus* section *Nigri* contamination plays a significant role in the production of mycotoxins, such as OTA, in grapes, posing a serious phytopathological problem, particularly for operators in the wine sector [20]. However, Logrieco et al. [21] reported that *Aspergillus* section *Nigri* isolates isolated from grapes produced fumonisin B_2_. Determining the mycoflora that produces mycotoxins in grapes and their products, and assessing the related risks, is critical for identifying potential contamination and maintaining high-quality standards in grape and wine production (Mašková et al., 2014 [22]). According to the European Community Regulation (2023), there is a limit of 8 μg/kg for OTA in “dried vine fruits (currants, raisins and sultanas)” and 2 μg/kg for OTA in grape juice, grape juice from concentrate, concentrated “grape juice, grape nectar, grape must and concentrated grape must, placed on the market for the final consumer” [23]. However, there is a lack of information and regulations about possible mycotoxins of other toxigenic molds found in grapes. Considering the changing climatic conditions, there may be changes in the mold species contaminating grapes and the secondary metabolites they produce. There is very limited information in the literature about the presence of toxigenic *Fusarium* species in grapes and the mycotoxin production of the species found [10,11,24]. In the literature, studies have focused on diseases caused by phytopathogenic *Fusarium* species, particularly in grapevine plants [25,26,27]. The mycobiota of grapes is very important to determine, as it serves as an indicator of mycotoxins in grapes and their products. Therefore, *Fusarium* species isolates from Çavuş and Karalahna grapes grown in Bozcaada, Türkiye’s third-largest island, were studied.

## 2. Materials and Methods

*Fusarium* isolates were obtained from grape berries grown in vineyards in two different locations (Çayır and Sulubahçe) on Bozcaada during field studies conducted in 2015 and 2016 [19]. Microfungus isolations from grape samples were performed as described by Özcan Ateş and Zorba [19]. After isolation, the isolates were initially cultivated on slant Potato Dextrose Agar (PDA) (213400, BD, Franklin Lakes, NJ, USA) and grown at 25 °C for 14 days. Subsequently, 5 mL of a 0.2% agar (214010, BD, Franklin Lakes, NJ, USA) +0.05% Tween 80 (822187, Merck, Darmstadt, Germany) spore solution was added and stored at +4 °C. For the study, these stock cultures were cultivated on PDA medium and incubated at 25 °C for 7 days [2,19]. The revived and pure isolates were then cultured on PDA medium and incubated at room temperature for 14 days under a daylight cycle. After incubation, they were examined using the wet preparation method in Petri dishes [28,29,30,31].

The isolates’ colony morphologies were determined after 7 days of incubation at 25 °C on PDA and Spezieller Nährstoffarmer Agar (SNA).

A ‘Dneasy Ultraclean Microbial Kit (Qiagen, Hilden, Germany)’ for fast and easy DNA isolation via spin filter extraction was used for DNA isolation from isolates grown in Malt Extract Agar (MEA) (CM0059, Oxoid, Basingstoke, UK) medium at 25 °C for 7 days. DNA isolation was performed according to the manufacturer’s instructions. The purity of the DNA samples was verified on an agarose gel. The PCR process utilized Translational Elongation Factor 1-alpha (*tef1*) and Βeta-Tubulin (*tub2*) primers (Table 1). For the PCR reaction, the mixture included 7.5 μL of PCR Mix, 0.2 μL of HotStart Taq Polymerase (5 U/μL), 2 μL of PCR Optimizer (Betaine 5 M), 1 μL of Forward Primer (5 pmol), 1 μL of Reverse Primer (5 pmol), 2 μL of PCR-Grade Water, and 1.5 μL of template DNA. The amplification reaction was conducted under the following conditions: 1 cycle of 95 °C for 10 min, followed by 35 cycles of 30 s at 95 °C, 45 s at 59 °C, and 40 s at 72 °C. After the PCR process, 5 μL of the PCR product was loaded onto a 2% agarose gel to visualize the amplicons prior to the PCR Clean-Up step.

Sequence analyses were performed to investigate the phylogenetic relationships of the 17 isolates identified within the scope of the study on *tef1* and *tub2* gene regions selected, in line with the data in the literature [28,29,30,31]. PCR products were first purified using the PCR cleaning step and the Seqline PCR Clean-Up kit (2931A, EYS Medikal, Sokak, Türkiye). Forward and reverse sequencing were then performed with the Applied Biosystems 3500/3500xl Genetic Analyzers and Data Collection Software v3.0 or v3.1. Broken reads originated from the primer binding points of the samples whose sequencing process was completed and were excised with the Bioedit v7.0.53 program [34]. After editing the sequence data of the isolates, the sequences were subjected to multiple alignment using MEGA v6. [35]. The correctness of the polymorphisms was checked, reference isolates were used in the evaluation of all the isolates within the scope of the study, and the results were also checked via NCBI-Blast-n (https://blast.ncbi.nlm.nih.gov/Blast.cgi?PROGRAM=blastn&PAGE_TYPE=BlastSearch&LINK_LOC=blasthome, accessed date: 27 March 2025) and FUSARIOID-ID (https://www.fusarium.org, accessed date: 27 March 2025) [36].

## 3. Results

**Figure 1:**
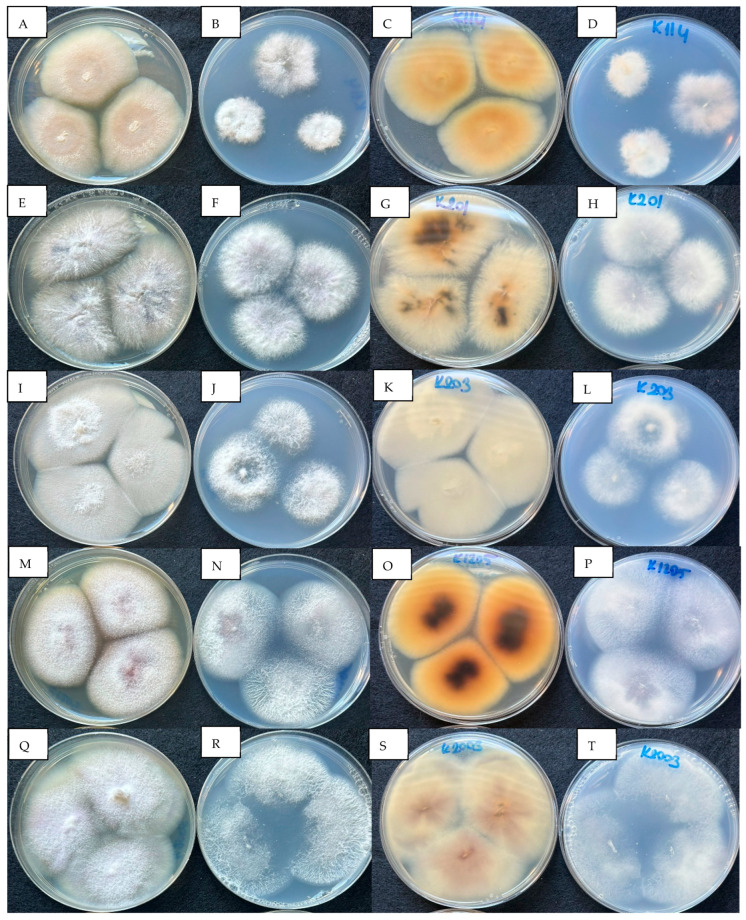
(**A**–**D**): K114 *F. makinsoniae* (possible synonym of *F. guilinense*) colonies grown at 25 °C for 7 days. (**A**). PDA. (**B**). SNA. (**C**). PDA reverse. (**D**). SNA reverse. (**E**–**H**): K201 *F. nirenbergiae* colonies grown at 25 °C for 7 days. (**E**). PDA. (**F**). SNA. (**G**). PDA reverse. (**H**). SNA reverse. (**I**–**L**): K203 *F. clavus* (as ‘*clavum’*) colonies grown at 25 °C for 7 days. (**I**). PDA. (**J**). SNA. (**K**). PDA reverse. (**L**). SNA reverse. (**M**–**P**): K1205 *F. annulatum* colonies grown at 25 °C for 7 days. (**M**). PDA. (**N**). SNA. (**O**). PDA reverse. (**P**). SNA reverse. (**Q**–**T**): K2003 *F. fabacearum* colonies grown at 25 °C for 7 days. (**Q**). PDA. (**R**). SNA. (**S**). PDA reverse. (**T**). SNA reverse.

In total, 17 *Fusarium* species were identified from Bozcaada Çavuş and Karalahna grapes using two gene regions. The identification of isolates was based on a BLASTn search of partial sequences. An NCBI BLASTn search was performed with nucleotide sequences of the *tef1* and *tub2* gene regions. The isolates were 98.97% to 100% similar to other *Fusarium* species in GenBank. However, the matches in the two gene regions did not show parallelism with the NCBI BLASTN matches. Therefore, nucleotide sequences were matched with the FUSARIOID-ID database by multi-locus sequencing, and it was determined that the matches were 98.71% to 100% similar. The identification with the NCBI Bank was made by considering a single-gene region of the isolates, and the similarities between the *tef1* and *tub2* genes are presented in Table 2. In the FUSARIOID-ID database, multiple sequence typing was performed with two gene regions, and the similarity rates are given in Table 3. The colony morphologies of the five isolates in the PDA and SNA media are shown in Figure 1.

**Figure 2:**
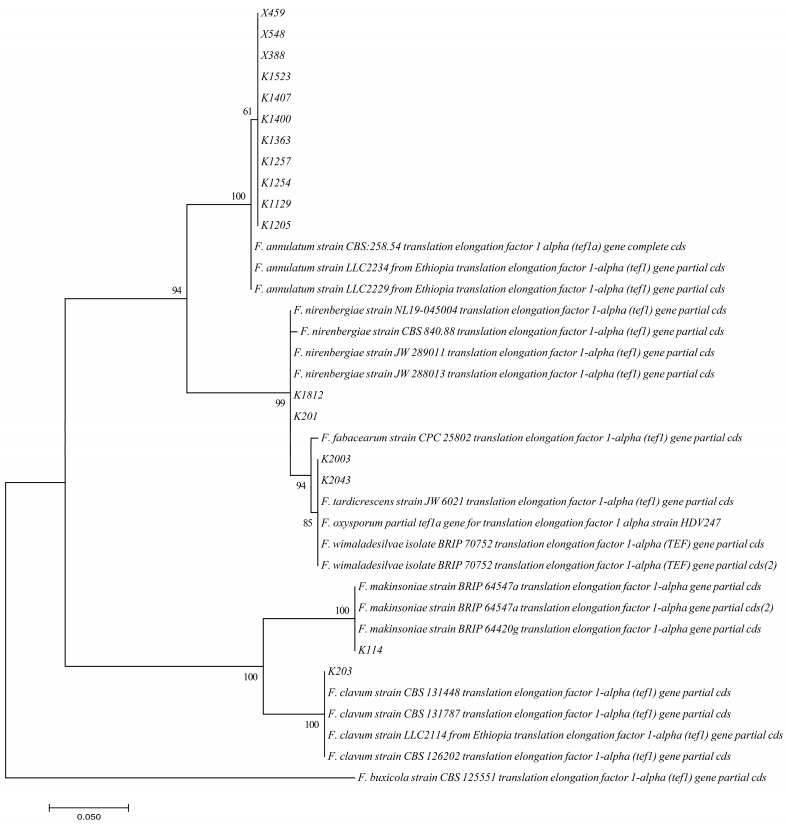
Maximum Likelihood Tree. Tamura–Nei model for *tef1* chosen for maximum likelihood analysis. Bootstrap set to 1000 replicas. Branch arrangements selected as TBR. KM231939.1 *Fusarium buxicola* strain CBS 125551 translation elongation factor 1-alpha (*tef1*) gene; partial cds used as outgroup.

The isolates were identified as *Fusarium makinsoniae* (1) and *Fusarium clavus* (as ‘*clavum’*) (1) from the *Fusarium incarnatum-equiseti* species complex (FIESC), *Fusarium nirenbergiae* (2) and *Fusarium fabacearum* (2) from the *Fusarium oxysporum* species complex (FOSC), and *Fusarium annulatum* (11) from the *Fusarium fujikuroi* species complex (FFSC). To construct the phylogenetic tree of the *tef1* gene, 20 reference sequences belonging to *Fusarium* species in GenBank were included. The *tef1* gene phylogenetic tree was rooted using *Fusarium buxicola* strain CBS 125551 (Figure 2).

## 4. Discussion

This study represents the first study on *Fusarium* species colonizing Bozcaada Çavuş and Karalahna grape berries. There are only two studies on the mycobiota and mycotoxigenic *Aspergillus* population of Bozcaada grapes and OTA production [2,19], with no other studies available. Thus, determining the mycobiota in agricultural products is significant, as it provides data on possible mycotoxin formation in that product [37].

There are limited studies on the isolation of *Fusarium* species from grapes. Mikušová et al. reported that the most frequently found species were *F. oxysporum*, *F. proliferatum*, *Fusarium semitectum*, *Fusarium solani*, *Fusarium subglutinans*, and *Fusarium verticillioides*, which they identified using traditional methods on Slovak grapes. They evaluated *Fusarium* species mycotoxins using HPLC-MS/MS. They found that *F. oxysporum* and *F. proliferatum*, which were grown on Czapek Yeast Autolysate Agar (CYA) and Yeast Extract Sucrose Agar (YES), produced beauvericin in amounts ranging from 3265 to 13,400 μg/kg and fusaproliferin in amounts ranging from 49,850 to 259,500 μg/kg [10,24]. Mašková et al. collected 24 samples of grapes used for wine production from various Slovak regions in 2012 and evaluated *Fusarium* isolates and their potential for producing mycotoxins. A total of 11 species were identified from grape samples, namely *Fusarium acuminatum*, *Fusarium avenaceum*, *F. graminearum*, *F. oxysporum*, *F. proliferatum*, *F. semitectum*, *F. solani*, *Fusarium sporotrichioides*, *F. subglutinans*, *Fusarium tricinctum*, and *F. verticillioides*. They reported that *F. proliferatum* and *F. sporotrichioides* were the most important species based on isolation frequency and relative density. They evaluated the mycotoxin production of selected isolates of these two species by thin-layer chromatography (TLC). As a result, only the sporadic production of diacetoxyscirpenol, HT-2, and T-2 toxins was confirmed by *F. proliferatum* isolates. In contrast, all the *F. sporotrichioides* isolates were capable of producing diacetoxyscirpenol, deoxynivalenol, and T-2 toxin; 73% of them were capable of producing HT-2 toxin, and 50% of them were capable of producing zearalenone [22]. Bolton et al. identified mycotoxigenic *Fusarium* species using molecular techniques targeting the *tef1* gene in grapes to assess mycotoxin risk in Southeastern American wine. They determined that *F. fujikuroi* was the most abundant species, followed by *F. proliferatum*, *F. incarnatum-equiseti*, *F. oxysporum*, *Fusarium concentricum*, and *F. solani*. They determined that *F. fujikuroi* and *F. proliferatum* isolates produced fumonisin B_1_, B_2_, and B_3_. They emphasized that the view that the *F. fujikuroi* species produces low fumonisin levels is incorrect. *Fusarium* species should be re-evaluated as a mycotoxinogenic threat to economically important crops [38]. Tančinová et al. identified *Fusarium* isolates obtained from Slovak wine grapes using traditional methods. They reported that the isolates were *F. acuminatum*, *F. avenaceum*, *Fusarium culmorum*, *F. equiseti*, *F. graminearum*, *F. oxysporum*, *F. proliferatum*, *F. semitectum*, *F. solani*, *F. sporotrichioides*, *F. subglutinans*, *F. tricinctum*, and *F. verticillioides*. When they evaluated the mycotoxin production of 47 isolates selected by random sampling using thin-layer chromatography, they determined that 68% produced at least one of the mycotoxins deoxynivalenol, T-2 toxin, HT-2 toxin, and diacetoxyscirpenol [11]. Cosseboom and Hu identified nine *Fusarium* isolates from grape bunches in Maryland, Pennsylvania, and Virginia wine vineyards with *tef1*-α. They identified seven as *F. fujikuroi*, one as *F. proliferatum* within the *F. fujikuroi* complex, and one as *F. graminearum* within the *Fusarium sambucinum* species complex [39]. In our study, *Fusarium* species diversity found in Çavuş and Karalahna grapes, which are specific to Bozcaada, was investigated with two gene regions, and as a result, *F. makinsoniae* (1) and *F. clavus* (as ’*clavum’*) (1) isolates from the FIESC, *F. nirenbergiae* (2) and *F. fabacearum* (2) isolates from the FOSC and *F. annulatum* (11) isolates from the FFSC were identified, and they are species that have not been previously detected in grape berries. While *F. annulatum*, *F. clavus*, and *F. nirenbergiae* species isolated in the study were previously identified in Türkiye [13], *F. makinsoniae* and *F. fabacearum* species are the first records for Türkiye [40,41,42].

There are studies in the literature on the presence of *F. annulatum* in grapevines [13,26,43], but no study has been performed on its detection in grape berries. *F. annulatum*, which is known to be a fumonisin producer [44], has also been reported to be a diacetoxyscirpenol, fusarenone-X, nivalenol, and neosolaniol producer by the LC-MS/MS of a *F. annulatum* isolate isolated from rice sheath rot [45]. FIESC members can also produce mycotoxins [26,46]. *F. nirenbergiae* has been reported to produce beauvericin and moniliformin [46]. Since the species isolated and identified in the study are those identified in the literature as mycotoxin producers, the infection of grapes with *Fusarium* spp. can pose a risk due to the wide range of mycotoxins produced by species belonging to this genus [47]. Furthermore, climate change today significantly affects the stages and rates of mycotoxin-producing fungal development, alters host resistance and host–pathogen interactions, and profoundly affects the conditions under which mycotoxin production occurs for each pathogen.

## 5. Conclusions

In conclusion, our study found that possibly mycotoxigenic *Fusarium* species were present in Bozcaada grapes. Compared to the studies in the literature, the diversity of isolated *Fusarium* species was different. Additionally, *F. makinsoniae* and *F. fabacearum* species were recorded, their first instances in Türkiye. Furthermore, the discovery of new combinations of mycotoxins, host plants, and geographical areas has drawn the attention of the scientific community and necessitated the development of new diagnostic tools and a deeper understanding of the biology and genetics of toxigenic fungi. Therefore, it is very important to identify mycotoxigenic species in grapes and determine the mycotoxin species they produce.

## Figures and Tables

**Table 1:** Primer sequences used in study.

Title 1	Abbreviations	Primer Sequence (5′–3′)	Reference
Translation Elongation Factor 1-alpha (*tef1*)	EF1-F	ATGGGTAAGGARGACAAGAC	[32]
	EF2-R	GGARGTACCAGTSATCATG	
Βeta-Tubulin (*tub2*)	T1-R	AACATGCGTGAGATTGTAAGT	[33]
	T22-R	TCTGGATGTTGTTGGGAATCC	

**Table 2:** Identification of *Fusarium* isolates determined by amplifying *tef1* and *tub2* gene and nucleotide sequences in NCBIblastn.

Isolate No.	*tef1*			*tub2*			Collection Date	Isolation Source	Location
	Closest Relative	Closest Accession Number	Identity (%)	Closest Relative	Closest Accession Number	Identity (%)			
K114	*F. makinsoniae*	OQ626867.1	100	*F. incarnatum*	MT895843.1	99.39	4 August 2015	Çavuş grape	Bozcaada Çayır
	*F.* cf. *incarnatum*	MW076638.1	100	*F.* cf. *‘incarnatum-equiseti’*	PQ435149.1	99.39			
	*F. makinsoniae*	OQ626866.1	100	*F.* cf. *‘incarnatum-equiseti’*	PQ360889.1	99.39			
				*F. incarnatum*	KP133060.1	99.39			
				*F.* cf. *‘incarnatum-equiseti’*	PQ360887.1	99.39			
				*F. incarnatum*	KP133061.1	99.39			
K201	*F. nirenbergiae*	PQ819571.1	100	*F. glycines*	OP642084.1	98.57	4 August 2015	Karalahna grape	Bozcaada Sulubahçe
	*F. oxysporum*	DQ016269.1	100	*F. oxysporum*	MH827997.1	98.57			
	*F. nirenbergiae*	PP431416.1	100	*F. oxysporum*	OP901509.1	98.57			
	*F. nirenbergiae*	MH485017.1	100	*F. oxysporum*	PP694827.1	98.57			
	*F. oxysporum* f. sp. *cepae*	EU220395.1	100	*F. oxysporum*	PQ435146.1	98.57			
	*F. oxysporum*	MF327613.1	100						
K203	*F. equiseti*	MK061540.1	100	*F. clavum*	PP796758.1	100	4 August 2015	Karalahna grape	Bozcaada Sulubahçe
	*F. clavum*	PP782146.1	100	*F. clavum*	ON292434.1	100			
	*F. equiseti*	KF651956.1	100	*F. clavum*	PP796769.	100			
	*Fusarium* sp. *NRRL 53091*	GU250573.1	100						
	*F. clavum*	ON292415.1	100						
K1129	*F. proliferatum*	PP928449.1	99.76	*F. fujikuroi*	MK784204.1	99.61	20 June 2016	Çavuş grape	Bozcaada Çayır
	*F. annulatum*	OQ925589.1	99.76	*F. fujikuroi*	MN896954.1	99.61			
	*F. proliferatum*	MT305198.1	99.76	*F. proliferatum*	MZ680574.1	99.61			
	*F. annulatum*	OK888534.1	99.76	*F. proliferatum*	ON152866.1	99.61			
	*F. proliferatum*	PP782632.1	99.76	*F. proliferatum*	JX174034.1	99.61			
K1205	*F. proliferatum*	PP928449.1	100	*F. fujikuroi*	MK784204.1	100	20 June 2016	Çavuş grape	Bozcaada Çayır
	*F. annulatum*	OQ925589.1	100	*F. fujikuroi*	MN896954.1	100			
	*F. proliferatum*	MT305198.1	100	*F. proliferatum*	MZ680574.1	100			
	*F. annulatum*	OK888534.1	100	*F. proliferatum*	LC171211.1	100			
	*F. proliferatum*	PP782632.1	100	*F. proliferatum*	ON152866.1	100			
K1254	*F. proliferatum*	PP928449.1	99.76	*F. fujikuroi*	MK784204.1	99.61	20 June 2016	Çavuş grape	Bozcaada Sulubahçe
	*F. annulatum*	OQ925589.1	99.76	*F. fujikuroi*	MN896954.1	99.61			
	*F. proliferatum*	MT305198.1	99.76	*F. proliferatum*	MZ680574.1	99.61			
	*F. annulatum*	OK888534.1	99.76	*F. proliferatum*	ON152866.1	99.61			
	*F. proliferatum*	PP782632.1	99.76						
K1257	*F. proliferatum*	PP928449.1	100	*F. fujikuroi*	MK784204.1	99.80	20 June 2016	Çavuş grape	Bozcaada Sulubahçe
	*F. annulatum*	OQ925589.1	100	*F. fujikuroi*	MN896954.1	99.80			
	*F. proliferatum*	MT305198.1	100	*F. proliferatum*	MZ680574.1	99.80			
	*F. annulatum*	OK888534.1	100	*F. proliferatum*	LC171211.1	99.80			
	*F. proliferatum*	PP782632.1	100	*F. proliferatum*	ON152866.1	99.80			
K1363	*F. proliferatum*	OP273535.1	99.75	*F. fujikuroi*	MK784204.1	99.61	30 June 2016	Çavuş grape	Bozcaada Sulubahçe
	*F. proliferatum*	PP928449.1	99.75	*F. fujikuroi*	MN896954.1	99.61			
	*F. annulatum*	OQ925589.1	99.75	*F. proliferatum*	MZ680574.1	99.61			
	*F. proliferatum*	MT305198.1	99.75	*F. proliferatum*	ON152866.1	99.61			
	*F. annulatum*	OK888534.1	99.75	*F. proliferatum*	JX174034.1	99.61			
K1400	*F. proliferatum*	PP928449.1	99.76	*F. fujikuroi*	MK784204.1	99.80	10 July 2016	Çavuş grape	Bozcaada Çayır
	*F. annulatum*	OQ925589.1	99.76	*F. fujikuroi*	MN896954.1	99.80			
	*F. proliferatum*	MT305198.1	99.76	*F. proliferatum*	MZ680574.1	99.80			
	*F. annulatum*	OK888534.1	99.76	*F. proliferatum*	LC171211.1	99.80			
	*F. proliferatum*	PP782632.1	99.76	*F. proliferatum*	ON152866.	99.80			
K1407	*F. proliferatum*	PP928449.1	98.97	*F. fujikuroi*	MK784204.1	99.80	10 July 2016	Çavuş grape	Bozcaada Çayır
	*F. proliferatum*	MT305198.1	98.97	*F. fujikuroi*	MN896954.1	99.80			
	*F. annulatum*	OK888534.1	98.97	*F. proliferatum*	MZ680574.1	99.80			
	*F. proliferatum*	PP782632.1	98.97	*F. proliferatum*	LC171211.1	99.80			
	*F. annulatum*	OQ925612.1	98.97	*F. proliferatum*	ON152866.1	99.80			
K1523	*F. proliferatum*	PP928449.1	100	*F. fujikuroi*	MK784204.1	100	10 July 2016	Çavuş grape	Bozcaada Çayır
	*F. annulatum*	OQ925589.1	100	*F. fujikuroi*	MN896954.1	100			
	*F. proliferatum*	MT305198.1	100	*F. proliferatum*	MZ680574.1	100			
	*F. annulatum*	OK888534.1	100	*F. proliferatum*	LC171211.1	100			
	*F. proliferatum*	PP782632.1	100	*F. proliferatum*	ON152866.1	100			
K1812	*F. nirenbergiae*	PQ819571.1	100	*F. oxysporum* f. sp. *conglutinans*	ON292451.1	99.81	1 August 2016	Karalahna grape	Bozcaada Çayır
	*F. oxysporum*	DQ016269.1	100	*F. avenaceum*	ON292477.1	99.81			
	*F. nirenbergiae*	PP431416.1	100	*F. oxysporum*	LC414363.1	99.81			
	*F. nirenbergiae*	MH485017.1	100	*F. oxysporum* f. sp. *conglutinans*	ON292481.1	99.81			
	*F. oxysporum* f. sp. *cepae*	EU220395.1	100	*F. nirenbergiae*	ON292493.1	99.81			
K2003	*F. oxysporum*	KP964863.1	100	*F. oxysporum* f. sp. *dianthi*	LT841229.1	99.80	11August 2016	Çavuş grape	Bozcaada Sulubahçe
	*F. oxysporum*	KF537337.1	100	*F. oxysporum*	MN451160.1	99.80			
	*F. oxysporum*	MT078506.1	100	*F. oxysporum* f. sp. *pisi*	KP964945.1	99.80			
	*F. oxysporum*	MK461970.1	100	*F. oxysporum* f. sp. *vasinfectum*	KT323796.1	99.80			
	*F. oxysporum*	OQ511060.1	100	*F. oxysporum*	MN451093.1	99.80			
K2043	*F. oxysporum*	KP964863.1	99.75	*F. oxysporum* f. sp. *dianthi*	LT841229.1	100	22 August 2016	Karalahna grape	Bozccada Sulubahçe
	*F. oxysporum*	KF537337.1	99.75	*F. oxysporum*	MN451160.1	100			
	*F. oxysporum*	MT078506.1	99.75	*F. oxysporum* f. sp. *pisi*	KP964945.1	100			
	*F. oxysporum*	MK461970.1	99.75	*F. oxysporum* f. sp. *vasinfectum*	KT323796.1	100			
	*F. oxysporum*	OQ511060.1	99.75	*F. oxysporum*	MN451093.1	100			
X388	*F. proliferatum*	PP928449.1	98.97	*F. fujikuroi*	MK784204.1	100	20 July 2016	Çavuş grape	Bozcaada Sulubahçe
	*F. proliferatum*	MT305198.1	98.97	*F. fujikuroi*	MN896954.1	100			
	*F. annulatum*	OK888534.1	98.97	*F. proliferatum*	MZ680574.1	100			
	*F. proliferatum*	PP782632.1	98.97	*F. proliferatum*	LC171211.1	100			
	*F. annulatum*	OQ92561	98.97	*F. proliferatum*	ON152866.1	100			
X459	*F. proliferatum*	PP928449.1	99.76	*F. fujikuroi*	MK784204.1	100	1 August 2016	Çavuş grape	Bozcaada Sulubahçe
	*F. annulatum*	OQ925589.1	99.76	*F. fujikuroi*	MN896954.1	100			
	*F. proliferatum*	MT305198.1	99.76	*F. proliferatum*	MZ680574.1	100			
	*F. annulatum*	OK888534.1	99.76	*F. proliferatum*	ON152866.1	100			
	*F. proliferatum*	PP782632.1	99.76	*F. proliferatum*	JX174034.1	100			
X548	*F. proliferatum*	PP928449.1	100	*F. fujikuroi*	MK784204.1	100	11 August 2016	Çavuş grape	Bozcaada Sulubahçe
	*F. annulatum*	OQ925589.1	100	*F. fujikuroi*	MN896954.1	100			
	*F. proliferatum*	MT305198.1	100	*F. proliferatum*	MZ680574.1	100			
	*F. annulatum*	OK888534.1	100	*F. proliferatum*	LC171211.1	100			
	*F. proliferatum*	PP782632.1	100	*F. proliferatum*	ON152866.1	100			

**Table 3:** Identification of *Fusarium* isolates identified by amplification of *tef1* and *tub2* gene nucleotide sequences with multi-locus in FUSARIOID-OD.

Isolate No.	Identity (%)	Closest Species	Strain Number
K114	100	*F. makinsoniae* (possible synonym of *F. guilinense*)	BRIP 64420g
	100	*F. makinsoniae* (possible synonym of *F. guilinense*)	BRIP 64547a
K201	100	*F. nirenbergiae*	JW 288013
	100	*F. nirenbergiae*	JW 289011
K203	100	*F. clavus* (as ‘*clavum’*)	CBS 131448
	100	*F. clavus* (as ‘*clavum’*)	CBS 131787
K1129	98.74	*F. annulatum*	CBS 258.54
K1205	99.36	*F. annulatum*	CBS 258.54
K1254	99.37	*F. annulatum*	CBS 258.54
K1257	99.37	*F. annulatum*	CBS 258.54
K1363	98.71	*F. annulatum*	CBS 258.54
K1400	99.03	*F. annulatum*	CBS 258.54
K1407	98.71	*F. annulatum*	CBS 258.54
K1523	99.36	*F. annulatum*	CBS 258.54
K1812	100	*F. nirenbergiae*	JW 288013
	100	*F. nirenbergiae*	JW 289011
K2003	100	*F. fabacearum*	JW 6021
	100	*F. fabacearum*	JW 6043
	100	*F. fabacearum*	NRRL 37622
	100	*F. fabacearum*	LLC1367
	100	*F. fabacearum*	LLC1387
	100	*F. fabacearum*	LLC1410
	100	*F. fabacearum*	LLC1536
	100	*F. fabacearum*	LLC1682
	100	*F. fabacearum*	LLC3388
	100	*F. fabacearum*	LLC3481
	100	*F. wimaladesilvae*	BRIP 70752a
K2043	100	*F. fabacearum*	JW 6021
	100	*F. fabacearum*	JW 6043
	100	*F. fabacearum*	NRRL 37622
	100	*F. fabacearum*	LLC1367
	100	*F. fabacearum*	LLC1387
	100	*F. fabacearum*	LLC1410
	100	*F. fabacearum*	LLC1536
	100	*F. fabacearum*	LLC1682
	100	*F. fabacearum*	LLC3388
	100	*F. fabacearum*	LLC3481
	100	*F. wimaladesilvae*	BRIP 70752a
X388	98.98	*F. annulatum*	CBS 258.54
X459	99.03	*F. annulatum*	CBS 258.54
X548	99.36	*F. annulatum*	CBS 258.54

## Data Availability

The original contributions presented in this study are included in the article. Further inquiries can be directed to the author.
